# Reduced Graphene Oxide-Coated Separator to Activate Dead Potassium for Efficient Potassium Batteries

**DOI:** 10.3390/ma15165505

**Published:** 2022-08-10

**Authors:** Liping Si, Jianyi Wang, Xijun Xu

**Affiliations:** 1 School of Materials Science and Hydrogen Energy, Foshan University, Foshan 528000, China; 2 School of Chemical Engineering and Light Industry, Guangdong University of Technology, Guangzhou 510006, China

**Keywords:** separator, reduced graphene oxide, potassium metal batteries, dead K

## Abstract

Potassium (K) metal batteries (KMBs) have the advantages of relatively low electric potential (−2.93 V), high specific capacity (687 mAh g^−1^), and low cost, which are highly appealing to manufacturers of portable electric products and vehicles. However, the large amounts of “dead K” caused by K dendrite growth and volumetric expansion can cause severe K metal anode deactivation. Here, a thin layer of conductive reduced graphene oxide (rGO) was coated on a GF separator (rGO@GF) to activate the generated dead K. Compared with the batteries adopting an original separator, those adopting a modified separator have significantly improved specific capacity and cycling stability. The life of full-cell of KMBs combining an rGO@GF separator with synthesized K_0.51_V_2_O_5_ is expected to exceed 400 cycles, with an initial capacity of 92 mAh g^−1^ at 0.5 A g^−1^ and an attenuation rate per cycle as low as 0.03%. Our work demonstrates that a composite separator of high conductivity is beneficial for high performance KMBs.

## 1. Introduction

Portable electronic devices, electric vehicles, and large-scale grid energy storage all have a huge demand for high-energy-density rechargeable batteries [1,2,3,4,5,6,7]. Over the past few decades, a variety of secondary batteries have been developed, such as lithium-ion, sodium-ion, zinc-ion, and potassium-ion (K^+^) batteries [8,9,10,11,12,13,14,15,16]. Among the diverse rechargeable battery systems, potassium metal batteries (KMBs) have attracted much attention due to their high theoretical energy density (685 mAh g^−1^) [17,18,19,20,21,22], low costs, and reduction potential, which have led them to be considered as a promising alternative to lithium-ion batteries [23,24,25,26,27]. However, the K metal negative electrode has suffered from the issues of dendrite growth upon plating/striping and volumetric expansion during cycling [28,29]. Furthermore, the constant fracturing and repairing of the vulnerable solid electrolyte interface (SEI) layer can cause an irreversible K loss [30]. As a result, the tiny K particles or filaments detached from the substrate become tightly wrapped by the electrically insulated SEI layer, leading to dead K [31]. Continuous side reactions and large amounts of dead K aggregations between the separator and anode can result in reduced capacity and cycling performance [32]. In addition, the increase in inactive dead K could result in poor electrochemical kinetics [33,34,35,36]. Consequently, advanced strategies for activating dead K are highly desirable for the commercial application and utilization rate of KMBs [37]. Numerous endeavors have been undertaken to implement an appropriate design for a KMB system, including the modification of the electrodes, electrolyte, and separator [1,23,38]. However, the relevant mechanism of using a separator to improve the electrochemical performance has attracted less attention [39,40,41,42,43].

Because migrating K^+^ and anions pass through the separator during the charging and discharging processes, the modified separator is considered to be an appealing candidate for regulating ion migration [44]. However, due to the low electrical conductivity of the separator, dead K easily aggravates the situation, eventually leading to battery deactivation. To activate dead K on the separator and improve the utilization rate of K metal, inserting a highly conductive, thin layer, such as rGO [45,46], between the anode and the separator was proposed for lithium batteries. rGO has high conductivity and a highly stratified 2D structure [47,48,49], enabling its direct filtration onto the separator without the use of adhesive agents [48,50,51,52,53]. Therefore, the utilization of an advanced composite separator could be an alternative pathway for KIMs.

This paper is the first to demonstrate that the resulting modified separator (rGO@GF separator) can be directly applied in KMBs. Moreover, in order to test the practicality of such batteries, a reconstructed high-capacity cathode of K_0.51_V_2_O_5_ was applied to construct KMB-based full cells [54]. This modified separator not only activates the dead K but also reduces the transfer distance of K^+^ and improves conductivity. Using such an anode-facing rGO@GF, the specific capacity and cycling stability can be significantly improved for KMB-based full cells, achieving 500 steady cycles as compared to 100 cycles for half-cell/symmetrical batteries.

## 2. Results

**Figure 1:**
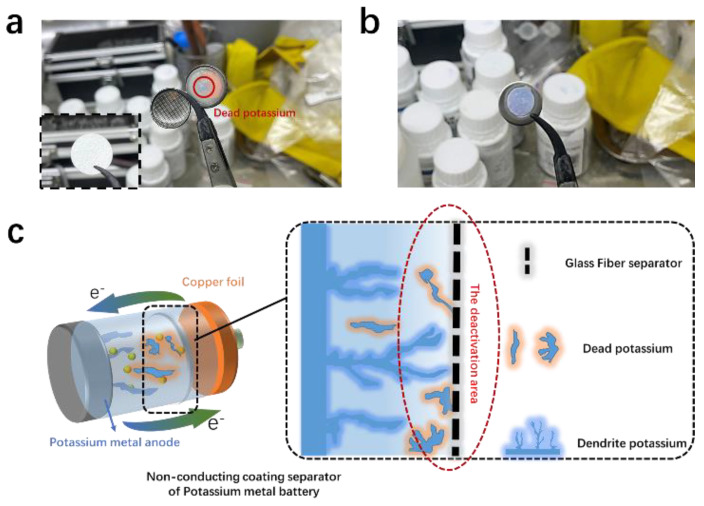
Photographs recording the dead potassium (K) on the glass fiber (GF) separator (**a**) and dendrite on K metal anode (**b**). (**c**) Schematic illustration of dendritic growth on K metal anode and formation of dead K, which adheres to the surface of the GF separator and gradually increases on the surface of the GF separator during charge/discharge processes.

To explore the effect of the GF separator on the formation of dead K, we first examined the conditions of dead K inside the conventional half-cell of KMBs. As shown in Figure 1, copper foil and a K metal sheet were used as the counter electrode. Driven by electrochemical kinetics, dendrite growth continuously extends from the interface (electrolyte/anode) toward the cathode. Theoretically, given the instability and brittleness of the SEI, cracks will continuously form on the original SEI, causing the rapid diffusion of K^+^ in regions around the cracks and the formation of new K dendrites. The repeated deposition/dissolution of K dendrites consumes the electrolyte and K metal, leading to the continuous aggregation of K around the anode. Therefore, constant morphological changes in K lead to the incapability to make direct contact with the anode, which causes the loss of electron contact. This condition is the fundamental reason for the formation of dead K (Figure 1a). Notably, most dendrites cannot pierce through the separator and are attached to its surface due to the relatively high hardness of GF separator. The part composed of K continuously cracks to form dead K, which exists around the anode region as an inactive substance, further slowing down the diffusion process. Some of the dead K is attached to the middle of the separator. The amount of dead K on the entire separator significantly increases, and many particles exist on the anode surface (Figure 1b), indicating that dead K is not only attached to the separator surface but also exists in the electrolyte in the form of suspended matter. Therefore, under most testing conditions, the dead K aggregates around the negative electrode region during the uneven dissolution process of K dendrites, leading to the rapidly reduced capacity of KMBs (Figure 1c). Given that dead K loses electron contact with current collectors, it is generally regarded as lacking electrochemical activity and thus not participating in subsequent battery reactions.

**Figure 2:**
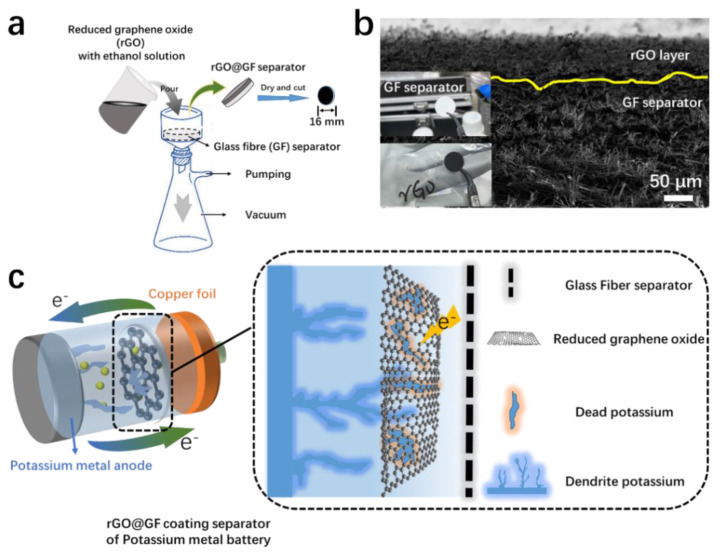
(**a**) Schematic of the fabrication of reduced graphene oxide on the glass fiber (rGO@GF) separator. (**b**) Surface morphologies of coated separator and photograph of pure GF separator and separator with an rGO@GF layer on the lower left. (**c**) Schematic illustration of rGO@GF separator working and activating dead potassium, inhibiting the K dendrite mechanism.

To sufficiently utilize the dead K on the separator surface, a conductive layer could be added to the separator surface. After filtering rGO through the separator, the resulting composite separator (rGO@GF separator) had a conductive interface (Figure 2a). The scanning electron microscopy (SEM, Figure 2b) image of rGO@GF showed a distinctly layered structure between rGO and GF. After the rGO was loaded, a dense nano-rGO layer formed on the GF surface as an overlying layer with a thickness of <1 µm and an area mass loading of 0.06 mg cm^−2^. The digital photo of the prepared rGO@GF separator is shown on the bottom left, and it can be observed that its surface shifted from white to black (Figure 2b), indicating that separator is uniformly covered by the rGO nanosheet (compared with the digital photo of pure separator). The dense black surface can ensure continuous electron transfer. The schematic of the rGO@GF separator for KMBs is shown in Figure 2c. The coating layer facing the K metal anode has electrochemical activity for dead K, and the reduction and oxidation reaction occurred on the rGO@GF layer. When a K dendrite contacts the conductive coating of rGO@GF, the dendrite is confined between the anode and modified separator, and this improves the utilization rate of the active material and the stability of the K metal anode.

**Figure 3:**
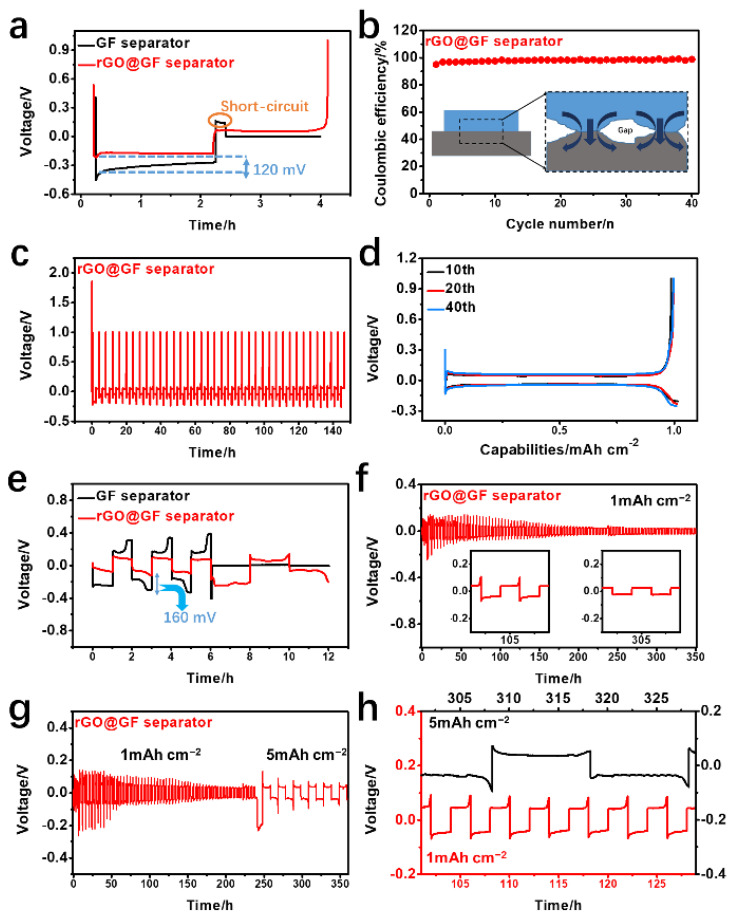
Electrochemical performance and surface state for potassium (K) metal anodes with conductive reduced graphene oxide or not. (**a**) First plating–stripping profiles of K/Cu battery with rGO@GF/GF separator. (**b**) Coulombic efficiency (CE) of K/Cu battery with rGO@GF separator of plating for 2 h with a current density of 0.5 mA cm^−2^ and stripping to 1 V. (**c**,**d**) Galvanostatic plating–stripping profiles for K/Cu battery with rGO@GF/GF separator. (**e**) Polarization of K symmetric battery with 0.1 to 1 mAh cm^−2^ on the rGO@GF/GF separator. (**f**) Polarization of K symmetric battery with long cycle at 1 mAh cm^−2^ on the rGO@GF separator. (**g**) Rate performance of K symmetric battery with area capacity of 1 and 5 mAh cm^−2^ on the rGO@GF separator, (**h**) compared with polarization of K symmetric battery with different area capacity.

The coulombic efficiency (CE), overall cycling stability, and voltage polarization of the rGO@GF separator applied to half and symmetric KMBs were first examined. Figure 3a shows a comparison of the cycling CEs of half batteries using a different separator under a current density of 0.5 mA cm^−2^. The half-cell assembled with the original separator could not be deeply charged. Without separator modification, the majority of the K was trapped in the anode area without being activated, and the SEI was continuously damaged by the ongoing cycling until the cycling itself could not continue anymore. Comparatively, the CE value of rGO@GF separator remained at 98% for 40 cycles (Figure 3b), indicating that the introduction of rGO to the anode region of KMBs through separator modification can alleviate dead K. The barrier layer of rGO can collect the dead K growing from the anode. Meanwhile, the rapid electron transfers on the rGO surface increased the kinetic transformation process of KMBs by innovatively using the conductive interface layer to form a second layer of current collectors, which can effectively reduce the transfer distance of K^+^ and enable K^+^ to freely move back and forth between the cathode and anode, thereby significantly reducing the transfer resistance of K^+^. From the SEM images of the half-cell after 40 cycles (Figure S1), it can be observed that the rGO@GF separator has a relatively even surface. The different morphology could be attributed to the 2D conductive structure of the rGO limiting the growth of K dendrites and reactivating dead K, allowing for the formation of a layer of stable K metal on the surface. This result indicates that the rGO@GF separator not only effectively activates dead K in KMBs but also increases the kinetics of the electrochemical process. Figure 3c shows the voltage changes of a half-cell based on an rGO@GF separator over time. These cycles indicate that half-cells of KMBs adopting rGO@GF separators have a stable voltage and a relatively small overpotential, suggesting that the K metal combined with the rGO@GF separator can compete with 3D current collectors without the need for protection. In addition, the long-cycle time–voltage and charge–discharge curves of 10th, 20th, and 40th cycles are shown in Figure 3d. The overpotential of the rGO@GF separator has a smooth voltage curve, indicating that conductive layer can carry out the stable stripping/plating of K metal. Studies of higher area capacities (5 mAh cm^−2^, Figure S2) have also shown that the rGO@GF separator has an equally good cycle efficiency, with a stable efficiency over 80% and cycling time over 200 h. This finding further indicates that rGO@GF not only significantly relieved the inactivation of dead K but also increased the utilization rate of K metal in KMBs.

As shown in Figure 3e, the cycling performances of different separators in symmetric-cell KMBs at a current density of 0.5 mA cm^−2^ were evaluated. None of the separators encountered micro-short-circuits in earlier cycles. Specifically, rGO@GF provided a symmetric-cell cycling time of up to about 350 h, which is longer than that of GF (10 h). The symmetric-cell voltage of the rGO@GF separator stabilized within 200 mV at an overpotential of 100–110 h and at 100 mV in the subsequent cycle, indicating that active dead K and K metal formed a stable SEI in the rGO@GF separator (Figure 3f). The time–voltage curves of rGO@GF separator with a regular and flat platform without tips or ridges in 300–310 h demonstrate that K deposition was easily induced on high-conductivity rGO, and the absence of voltage lag proves that the K metal anode was highly reversible. This results further indicates that the layer of rGO@GF promoted the recycling and activation of dead K, was conducive to reducing electrode polarization, and thus increased the cycling stability. More importantly, the rGO@GF separator also shows excellent rate performance (Figure 3g), with stable voltage polarization vibration at different area capabilities of 1.0–5.0 mAh cm^−2^ (Figure 3h), which further indicates that the rGO@GF separator has excellent interface stability. The rGO-coated separator can activate dead K on the surface of the separator and avoid the occurrence of an adverse reaction, which further helps to form a stable and dense SEI layer, homogenize K^+^ flux, and guide the uniform deposition of K that avoids the generation of dendrites, which effectively improves the stability of the K metal anode. In addition, the rGO@GF separator is equivalent or superior to similar reports from the literature in terms of areal capacity, cycle life, and current density.

**Figure 4:**
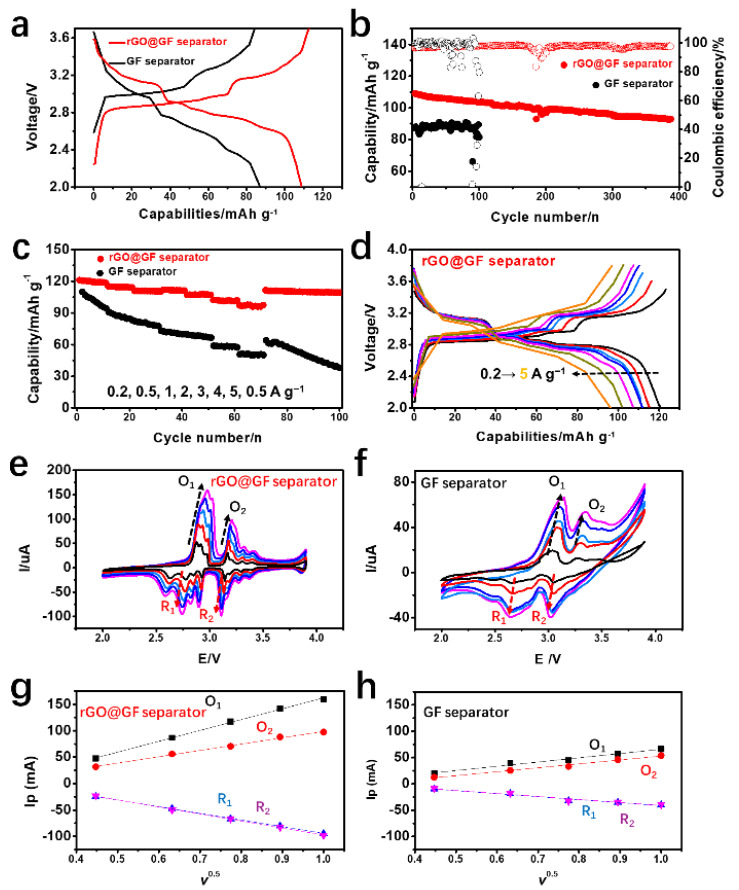
Electrochemical performance comparison of full cells with K_0.51_V_2_O_5_ cathodes by using rGO@GF/GF separator. Comparison at the current density of 0.5 A g^−1^: (**a**) charging–discharging curve of first cycle, (**b**) cycling performance, (**c**) rate performance of rGO@GF/GF separator, and (**d**) whole charging–discharging profiles of rGO@GF separator. (**e**,**f**) Comparison of kinetic behaviors of CV curves at multiple scan rates (0.2, 0.4, 0.6, 0.8, and 1 mV s^−1^). (**g**,**h**) Comparison of the linear fits of the peak currents for KMBs with different separators.

Full-cell testing of KMBs is the most important step in testing the practicality of such batteries. The performance of KMBs depends not only on the structure and performance of the cathode and anode materials but also on the separator. The development of a multi-functional separator is expected to overcome many difficulties encountered in the research on practical KMBs. The effect of the rGO@GF separator on KMB performance was tested by combining a K metal anode and a cathode composed of K_0.51_V_2_O_5_. To compare the rGO@GF and GF separators on an equal basis, we considered the quality of the rGO coating. The electrochemical performance of the combination of the 80 wt% K_0.51_V_2_O_5_ and the rGO@GF separator and that of the 70 wt% K_0.51_V_2_O_5_ and GF separator were compared. Figure 4a shows the first charging and discharging curve of the KMBs adopting different separators at 0.5 A g^−1^. Both KMBs exhibited the typical voltage distributions of K_0.51_V_2_O_5_ batteries [54]. The battery adopting the rGO@GF separator had a longer voltage plateau than that adopting the GF separator, indicating that the rGO@GF separator can improve electrochemical performance. The initial discharging capacity of the former was up to 108 mAh g^−1^, which is 21 mAh g^−1^ higher than that of the GF-based battery. The conductive carbon coating on the rGO@GF surface increased the conductive area, thus increasing the conductivity of K^+^ and the utilization rate of dead K on the separator. Figure S3 shows the cyclic voltammetry (CV) of the rGO@GF and GF separators at 0.2 mV s^−1^, which is consistent with the results shown in the charge–discharge curve, and the voltage polarization is smaller, indicating that the battery using the rGO@GF separator as a conductive coating has excellent stability and reversibility. Figure 4b shows the cycling stability of the batteries adopting different separators. After 92 cycles, the capacity of the GF rapidly attenuated to 66 mAh g^−1^; the fast capacity attenuation exhibited by KMBs adopting a GF separator may be attributed to the unstable electrochemical cycles caused by the constant generation of dead K. Comparatively, the rGO@GF separator can maintain a capacity of 104 mAh g^−1^. The specific capacity and attenuation rates after 200 equivalent cycles were 92 mAh g^−1^ and 0.03%, respectively. This result indicates that KMBs with the rGO@GF separator have superior electrochemical performance compared with those adopting the original GF separator. In general, the specific capacity of the active material dropped along with an increase in the weight percentage in electrodes, indicating that the additional K metal deposited on the anode surface accelerated the generation of dead K. The 3D current collector structure adopting porous meshes and having high specific surface area can reduce the local current density and thus inhibit the growth of K dendrites and volumetric expansion in cycling. However, building low-cost, high-performance KMBs is still challenging. This condition is consistent with the activation mechanism of applying a conductive carbon layer to our proposed separator. Figure S4 shows the voltage-specific capacity diagram for the 100th, 200th and 300th cycles of the full-cell KMBs. It is worth noting that the charge–discharge curves were highly coincident, indicating that the K metal anode with the rGO@GF separator is highly reversible. All electrochemical results indicated that the rGO conductive carbon layer on the separator improved the cycling stability of batteries regardless of whether the 3D current collectors were added to the negative electrode. The current rate (A g^−1^ rate) of the full cell is also an important parameter. Under different current densities at 0.2–5 A g^−1^, as shown in Figure 4c,d, the rate test of KMBs based on the modified rGO@GF separator still showed a superior cycling performance under a large current of 5 A g^−1^. In particular, under rate cycling at 5 C, rGO@GF showed a high capacity of 96 mAh g^−1^, which further indicates that the rGO@GF separator promises the attainment of a stable SEI film and fast charge transfer.

From the charge–discharge curves of different current densities (Figure 4d), the excellent cycling performance of the rGO@GF separator is evident, and it can be concluded that its cycling performance is still good at a higher current density. This phenomenon can be explained by the diffusion kinetics of K^+^ in KMBs, according to the diffusion formula τ = L^2^/D, where τ is the diffusion time of K^+^ in KMBs, L is the diffusion distance of K^+^, and D is the chemical diffusion coefficient of K^+^. When the current density is lower, the diffusion time of K^+^ is longer than that at high current density, and the diffusion distance in KMBs is correspondingly longer. Therefore, the specific capacities of the rGO@GF and GF separators are higher at low current density (Figure S5). However, the fast K^+^ diffusion rate leads to K metal degradation and K metal anode volume expansion being greater during the cycle, resulting in a decrease in the specific capacity of the electrode in subsequent cycles. Therefore, in the case of high current density, due to the short diffusion distance, the plating/stripping of K occurs more quickly, which makes it difficult for the electrode to maintain good structural stability. Therefore, the rGO@GF separator with the short migration distance has an obvious advantage. Although the discharge specific capacity is smaller under low current density, the rGO@GF separator for KMBs is more stable than the GF separator.

As discussed earlier, the activation of dead K plays a crucial role in the improvement of the electrochemical performance of KMBs. Therefore, the diffusion characteristics of the separator must be evaluated. However, the coefficient of diffusivity of the separator is difficult to obtain through conventional measurements. Notably, a CV kinetic analysis can be performed to calculate the diffusivity of K^+^ and reveal its diffusion across different separators (Figure 4e,f). A high K^+^ diffusivity promotes the electrochemical reaction and controllable deposition of K^+^, maintains the activity of K metal anodes, and ensures the superior cycling performance of batteries. Therefore, the diffusivity of K^+^ can be regarded as an effective indicator of electrochemical transformation. Based on the CV curves under different scanning rates (0.2, 0.4, 0.6, 0.8, and 1 mV s^−1^), the *D*_K_^+^ values (Figure 4g,h), which were around 2.9/2.7 (R_1_/O_1_) and 3.2/3.1 V (R_2_/O_2_), can be calculated using the Randles–Sevcik equation [55]:

$$I_{p}=2.69\times 10^{5}n^{1.5}AD_{K^{+}}{\;}^{0.5}C_{K^{+}}v^{0.5}$$

In the GF-based KMBs, the *D*_K_^+^ (R_1_/O_1_) and *D*_K_^+^ (R_2_/O_2_) were 1.6 × 10^−8^/1.2 × 10^−8^ and 2.2 × 10^−8^/1.2 × 10^−8^ cm^2^·s^−1^, respectively. In the rGO@GF-separator-based KMBs, the *D*_K_^+^ (R_1_/O_1_) and *D*_K_^+^ (R_2_/O_2_) were 1.6 × 10^−7^/2.6 × 10^−8^ and 5.7 × 10^−8^/2.7 × 10^−9^ cm^2^·s^−1^, respectively. The significantly increased *D*_K_^+^ demonstrated that rGO@GF reduced the resistance to ion diffusion and promoted the redox transformation of dead K. In addition, the intensity of redox peaks of batteries adopting rGO@GF significantly increased, confirming that the rGO conductive carbon layer can significantly promote the electrochemical performance of KMBs. Furthermore, the reduction and oxidation peaks of the CV curve of the rGO@GF separator under different scanning rates showed positive and negative offsets, respectively, further indicating rapid electrochemical redox kinetics.

**Figure 5:**
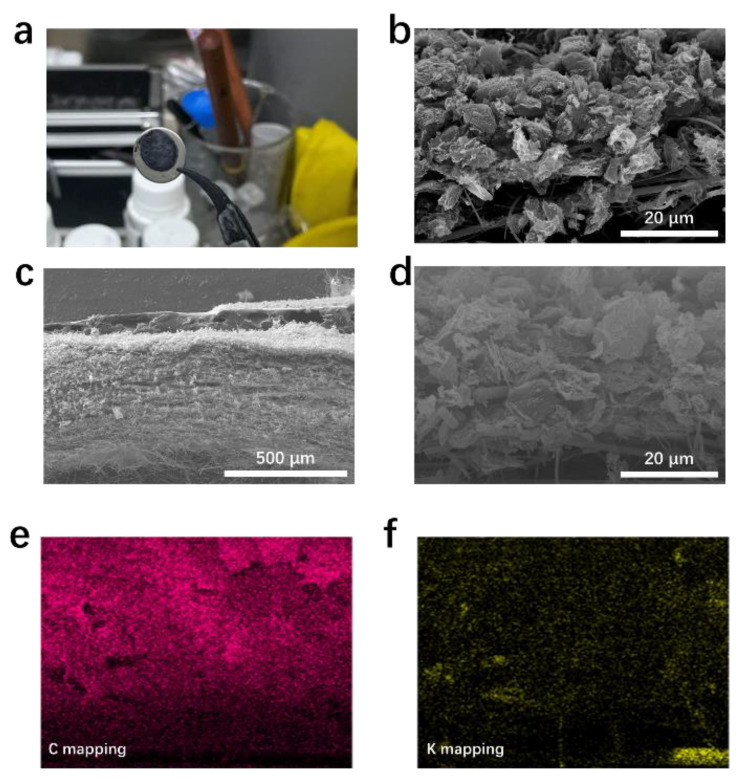
(**a**) Optical photograph of separator for K metal anode after cycles at 0.5 A g^−1^ with rGO@GF separator; top (**b**) and side (**c**,**d**) view of SEM and mapping (**e**,**f**) images of cycled rGO@GF separator.

To further prove the activating effect of the rGO conductive carbon layer on dead K on the separator, the rGO@GF separator was taken from a K metal half-cell after its 50th cycle was characterized. Figure 5a–d shows that the color of the rGO@GF separator and cycled K metal anode was relatively smooth, indicating that the majority of dead K that detached from the anode was attached to the rGO surface. The distribution of elements at the cycled rGO@GF separator (Figure 5e,f) was analyzed by using energy-spectrum scanning as shown in Figure 5c, which shows that enrichment of K and C exists on the layer of rGO. Figure S6 shows that according to XPS in-depth analysis of the surface of the cycled separator, the SEI is mainly composed of complex fluorine/oxygen-containing organic and inorganic compounds. The content of KF of the SEI of the rGO@GF separator is much higher than that of the GF separator, indicating that the SEI is rich in inorganic substances [39]. In addition, the signal of KF of the GF separator is lower, indicating that its SEI layer is thinner, reduces K and electrolyte consumption, and enables higher CE, lower resistance, and faster interfacial K^+^ transfer.

## 3. Discussion

**Figure 6:**
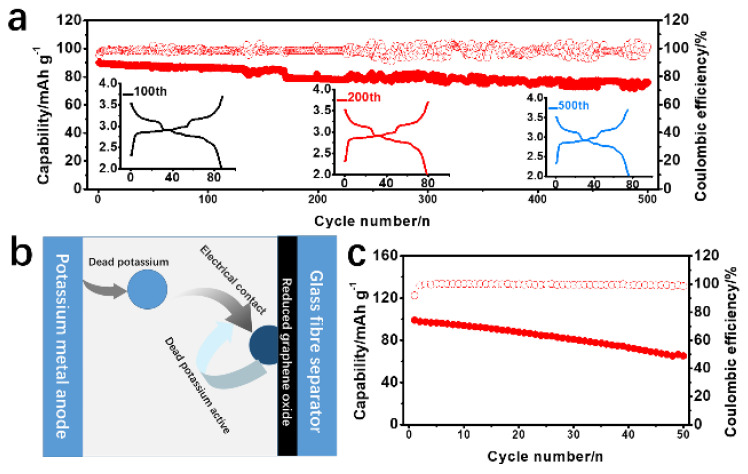
Electrochemical performance of full cells with failed potassium (K) metal anodes by using rGO@GF separator, (**a**) long-cycled with electrolyte of 3 M KFSI/DME and charging–discharging curve on the bottom. (**b**) Schematic illustration of rGO@GF separator activating failed K metal anode in KMB, (**c**) cycled with electrolyte of 0.8 M KPF_6_/EC/DMC.

**Figure 7:**
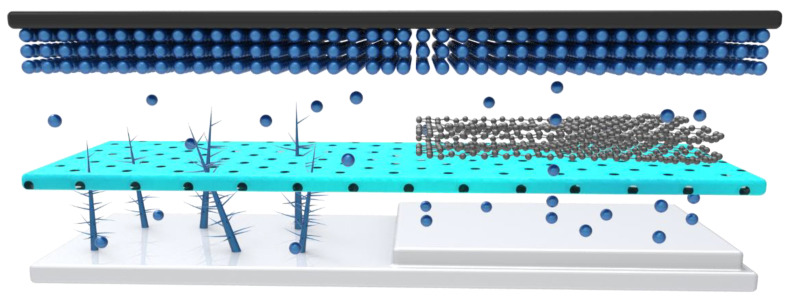
A diagram to illustrate the mechanism of rGO improving the battery performance.

In order to verify the reactivation performance of the modified separator, a failed K metal anode was assembled with the rGO@GF separator and a new electrolyte, which again showed a capacity of 90 mAh g^−1^ and a lower voltage polarization (Figure 6a), further demonstrating that the new rGO@GF separator can stabilize a dead K metal anode. The discharge capacity reached up to 73 mAh g^−1^ after 500th cycle, and the capacity retention rate was 79%. The schematic diagram in Figure 6b illustrates the good reversibility of the modified rGO@GF separator. rGO can transport the electrochemical reaction of KK^+^ to the surface of the rGO@GF separator through contact with dead K and can be reused during the charging process. The electrochemical performance of the ultra-long cycle indicates that the reaction dynamics are enhanced, which may explain its excellent performance. Also, to check whether this modified separator is suitable for other electrolyte system, an ester-type electrolyte (0.8 M KPF_6_/EC = ethylene carbonate/DEC = diethyl carbonate) was also investigated, as shown in Figure 6c; a considerable capacity was restored in that case as well. This suggested that the modified separator has a positive effect on and application prospect for activating dead K and inhibiting dendrites of K. The abundant nanochannel structures in rGO are beneficial to the migration of K^+^, and the gap channels can realize strong electrostatic repulsion of anions, thus limiting the free migration of anions. After laying an rGO layer onto a GF separator, the amount of K^+^ migration was increased, indicating that the migration of K^+^ was improved and the migration of anions was limited. In addition, a relatively uniform rGO coating has rich nanochannels, which can achieve uniform K deposition, thereby further inhibiting the growth process of K dendrites. Therefore, a highly stable K plating/stripping process was achieved by using the rGO@GF separator. In addition, the electrochemical performance of KMBs prepared with the rGO@GF separator was improved compared to the GF separator (Figure 7). The battery showed extremely stable cycle performance in more than 500 cycles, with a capacity retention rate of 81% and an average CE of 98%, which exceeded the corresponding values of most reported KMBs. This study demonstrates the potential role of rGO in regulating ion migration to achieve high-rate and ultra-long-cycle KMBs [43,56,57,58,59,60]. In addition, good capacity retention and recovery characteristics can still be obtained when an ester electrolyte is matched with a cycled K anode and a modified separator [37], a design which shows great potential for production at scale and good commercial development prospects.

## 4. Conclusions

In conclusion, we proposed an appropriate design to improve KMB performance by coating the separator with a thin rGO conductive layer. The special 2D structure of rGO not only inhibited K dendrites from piercing through the separator but also activated the attached dead K to improve the utilization rate of KMBs. Our research showed that modifying the most crucial separator in KMBs can effectively improve the electrochemical performance of KMBs, and the separator-surface-modification method opens up a new avenue for preparing high-performance KMBs.

## Data Availability

The data that support the findings of this study are available from the corresponding author upon reasonable request.

## Supplementary Materials

The following supporting information can be downloaded at https://www.mdpi.com/article/10.3390/ma15165505/s1: Materials and Methods, Figures S1–S6. Figure S1: Top-down morphology K metal surfaces of cycled of rGO separator. Figure S2: Galvanostatic plating–stripping profiles for K/Cu battery with rGO@GF separator at ultra-high area capacity of 5 mAh cm^−2^ (a,b). Figure S3: Multiple charging–discharging curve of KMBs full-cell with rGO@GF separator at 0.5 A g^−1^. Figure S4: Comparison at kinetic behaviors of CV curves at 0.2 mV s^−1^ with rGO@GF (a) and rGO (b) separator. Figure S5: charging–discharging profiles of GF separator at 0.2, 0.5, 1, 2, 3, 4 and 5 A g^−1^. Figure S6: X-ray photoelectron spectrometer (XPS) results of C 1s, K 2p, F 1s, O 1s with cycled rGO@GF separator and GF separator.
