# Supplementary material for: Reduced Graphene Oxide-Coated Separator to Activate Dead Potassium for Efficient Potassium Batteries
Source: Materials (Basel). 2022 Aug 10;15(16):5505. doi: 10.3390/ma15165505 (PMC9412676; doi:10.3390/ma15165505)
Supplement: Supplementary file 1 [file materials-15-05505-s001.zip › materials-1809243-supplementary.pdf]

materials

Supporting Information

Reduced Graphene Oxide-Coated Separator to Activate Dead Potassium for Efficient Potassium Batteries

Liping Si [1], Jianyi Wang [1,*] and Xijun Xu [2,*]

1 School of Materials Science and Hydrogen Energy, Foshan University, Foshan 528000, China
2 School of Chemical Engineering and Light Industry, Guangdong University of Technology, Guangzhou 510006, China
* Correspondence: energywang@foxmail.com (J.W.); xuxijun2019@scut.edu.cn (X.X.)

EXPERIMENTAL SECTION

Preparation of GO@GF separator

Layered reduced graphene oxide (rGO, NO-XR021, 3-10 nm) was purchased from Nanjing Xianfeng Nanomaterials Technology Co., Ltd., the purchased of rGO is dispersed into ethanol （Aladdin, 99.5%） solution without purification and ultrasonic dispersion forms a turbid graphene solution. The rGO was uniformly loaded on one side of the Glass fiber separator (GF, Whatman GF/A1820-090) to form a composite (rGO@GF) separator. Dry the wet rGO@GF in a 60 °C vacuum oven for a night. Finally, the rGO@GF separator is perforated into a 16mm diameter disc.

Preparation of potassium cathode ($K_{0.51}V_2O_5$)

2mmol V_2O_5 (Aladdin), 2mmol KI (Aladdin) and 4mmol KCl (Aladdin) were dispersed in 50mL deionized water and stirred vigorously for half of a hour. Then, the mixed solution was transferred to a reaction kettle with a volume capacity of 100mL, and the kettle was sealed at 200℃ and heated for 24 h. Finally, the obtained products were collected by centrifugation, distilled water was used as detergent, and dried in a vacuum oven at 60℃ for 12 hours to obtain potassium cathode material ($K_{0.51}V_2O_5$). By mixing $K_{0.51}V_2O_5$, Super P and PVDF in NMP solution in proportion, stirring for 5h. The slurry was smeared on carbon-coated aluminum foil with a scraper and dried in a vacuum oven at 60℃ for a night. The loading capacity in the cathode material is 1~2 mg cm^{-2}.

Preparation of potassium metal anode

In the full of argon glove box, take a small piece of potassium block from kerosene, Remover the surface kerosene with clean paper, cut off the blue and black oxides on the surface of potassium block, roll them out into thin slices with a thickness of 1~2mm, and cut them into round slices with a diameter of about 12mm with a mold.

Battery test

In order to evaluate the electrochemical performance of the modified separator, potassium metal was used as a control electrode. The electrolyte is 3M KFSI/DME. Assembly was carried out in a model 2032 button battery mode in a glove box filled with argon gas, and constant current cycling of the battery was carried out using a LANHE battery tester. Cyclic voltammetry data were obtained from Chenhua electrochemical workstation with a voltage range of 2-3.9 V.

Characteristic

The morphology and size of the samples were determined by scanning electron microscope (SEM, JEOL), and the element distribution of the samples was determined by X-ray energy dispersive spectrometer (EDX, Oxford). The elemental composition and valence states of the samples were determined by X-ray photoelectron spectroscopy (XPS, ESCALAB 250 X-ray).

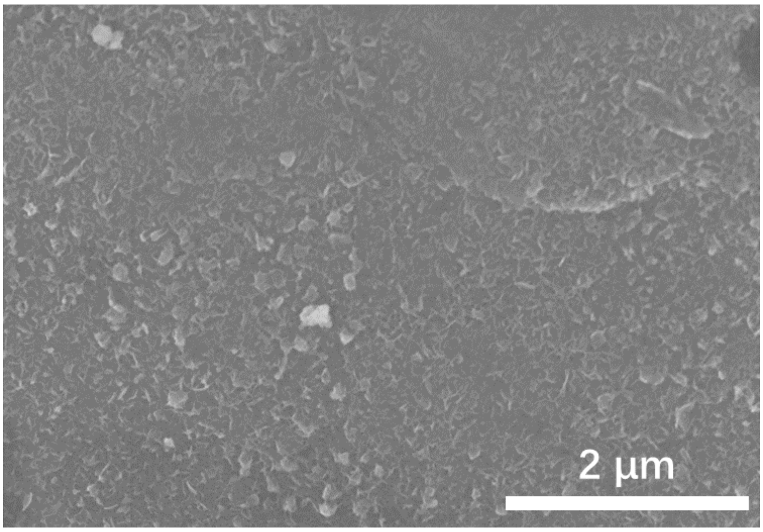

Figure S1. Top-down morphology K metal surfaces of cycled of rGO separator.

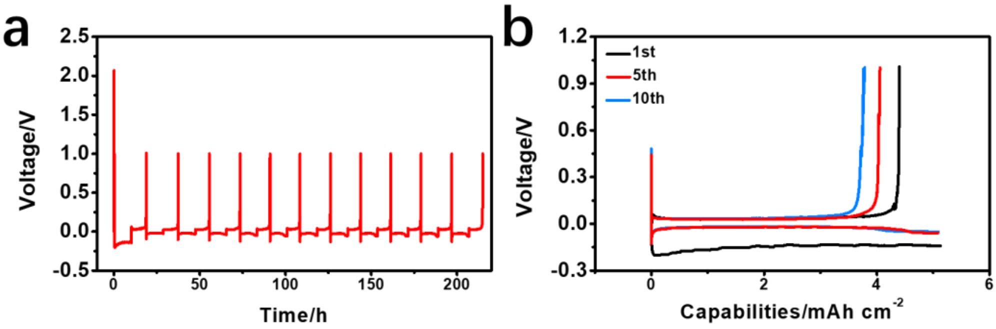

Figure S2. Galvanostatic plating–stripping profiles for K/Cu battery with rGO@GF separator at ultra-high area capacity of 5 mAh cm^{-2} (a,b).

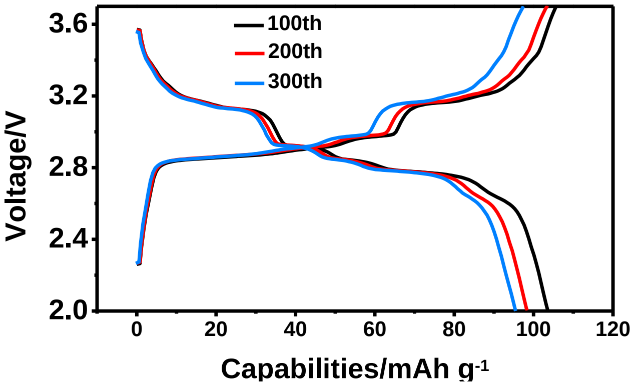

Figure S3. Multiple charging–discharging curve of KMBs full-cell with rGO@GF separator at 0.5 A g^{-1}.

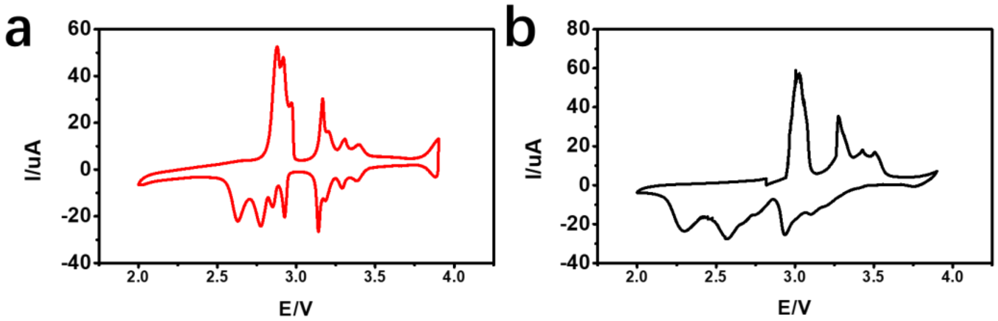

Figure S4. Comparison at kinetic behaviors of CV curves at 0.2 mV s-1 with rGO@GF (a) and rGO (b) separator.

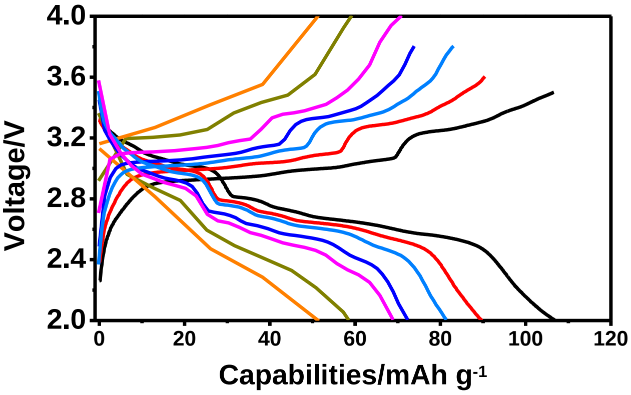

Figure S5. charging–discharging profiles of GF separator at 0.2, 0.5, 1, 2, 3, 4 and 5 A g^{-1}.

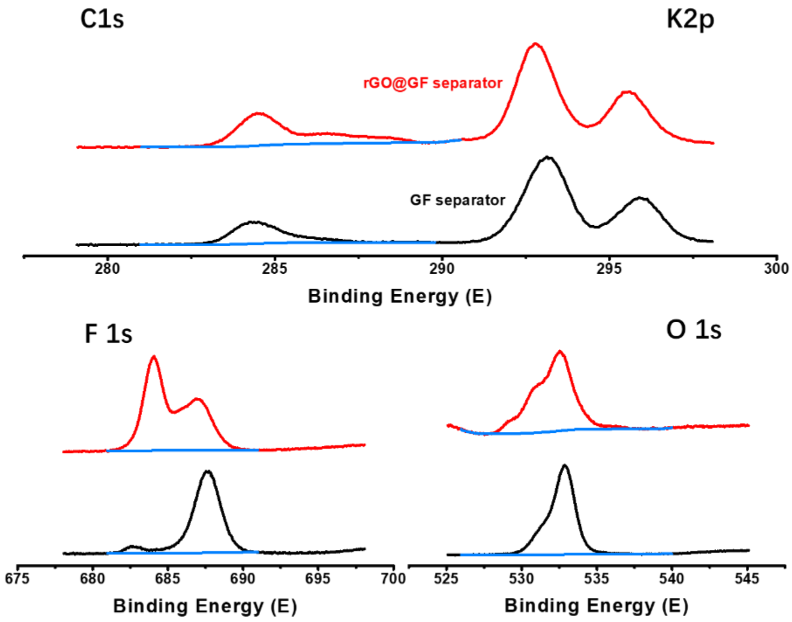

Figure S6. X-ray photoelectron spectrometer (XPS) results of C 1s, K 2p, F 1s, O 1s with cycled rGO@GF separator and GF separator.
